# SPRED2: A Novel Regulator of Epithelial-Mesenchymal Transition and Stemness in Hepatocellular Carcinoma Cells

**DOI:** 10.3390/ijms24054996

**Published:** 2023-03-05

**Authors:** Tong Gao, Xu Yang, Masayoshi Fujisawa, Toshiaki Ohara, Tianyi Wang, Nahoko Tomonobu, Masakiyo Sakaguchi, Teizo Yoshimura, Akihiro Matsukawa

**Affiliations:** 1 Department of Pathology and Experimental Medicine, Graduate School of Medicine, Dentistry and Pharmaceutical Sciences, Okayama University, Okayama 700-8558, Japan; 2 Department of Cell Biology, Graduate School of Medicine, Dentistry and Pharmaceutical Sciences, Okayama University, Okayama 700-8558, Japan

**Keywords:** cancer stem cells, epithelial–mesenchymal transition, ERK1/2-MAPK, tumorigenesis

## Abstract

The downregulation of SPRED2, a negative regulator of the ERK1/2 pathway, was previously detected in human cancers; however, the biological consequence remains unknown. Here, we investigated the effects of SPRED2 loss on hepatocellular carcinoma (HCC) cell function. Human HCC cell lines, expressing various levels of SPRED2 and SPRED2 knockdown, increased ERK1/2 activation. SPRED2-knockout (KO)-HepG2 cells displayed an elongated spindle shape with increased cell migration/invasion and cadherin switching, with features of epithelial–mesenchymal transition (EMT). SPRED2-KO cells demonstrated a higher ability to form spheres and colonies, expressed higher levels of stemness markers and were more resistant to cisplatin. Interestingly, SPRED2-KO cells also expressed higher levels of the stem cell surface markers CD44 and CD90. When CD44^+^CD90^+^ and CD44^−^CD90^−^ populations from WT cells were analyzed, a lower level of SPRED2 and higher levels of stem cell markers were detected in CD44^+^CD90^+^ cells. Further, endogenous SPRED2 expression decreased when WT cells were cultured in 3D, but was restored in 2D culture. Finally, the levels of SPRED2 in clinical HCC tissues were significantly lower than those in adjacent non-HCC tissues and were negatively associated with progression-free survival. Thus, the downregulation of SPRED2 in HCC promotes EMT and stemness through the activation of the ERK1/2 pathway, and leads to more malignant phenotypes.

## 1. Introduction

Liver cancer ranks 6th in incidence and 4th in mortality worldwide [1]. Although overall cancer death rates have decreased, the 5-year survival rate of liver cancer patients is 18% and liver cancer is the second most lethal cancer after pancreatic cancer [2]; thus, liver cancer remains a global health challenge. Approximately 70–90% of liver cancers are hepatocellular carcinoma (HCC), resulting from multiple etiologic landscapes, including viral infection, alcohol abuse, metabolic syndrome, obesity, and genetic alteration [3,4]. Recently, much attention has been directed to the contribution of the ERK1/2 pathway to HCC carcinogenesis [4]. Aberrant activation of the ERK1/2 pathway is frequently observed in human HCC [5,6]. Sorafenib, a Raf-1 kinase inhibitor, was the first systemic drug approved by the FDA for the treatment of advanced HCC; it improved the overall survival rate and delayed the time to progression [7], indicating that the ERK1/2 pathway is a major driver in HCC.

SPRED2 (Sprouty-related, EVH1 domain-containing protein 2) is a member of the SPRED protein family and inhibits Ras-dependent ERK1/2 signaling by suppressing the phosphorylation and activation of Raf [8]. The downregulation of SPRED2 expression was detected in advanced human cancers, including HCC [9]. Since the ERK1/2 pathway is a major driver in HCC [10], increased ERK1/2 activation by downregulated SPRED2 expression could contribute to the HCC development. We previously showed, using Spred2 (the term in mice)-deficient mice, that endogenous Spred2 controls the severity of several inflammatory diseases by downregulating the ERK1/2 pathway [11,12,13,14,15,16,17,18,19,20]. In a mouse model of bleomycin-induced lung injury [21], the strong accumulation of *Spred2* mRNA was observed in bronchial basal cells and club cells, which are tissue specific stem/progenitor cells [22]. The proliferation of bronchial basal cells and club cells was increased in bleomycin-treated mice with Spred2 deficiency [21]. These results suggest a role of endogenous SPRED2 in regulating the proliferation of stem cells. It is possible that downregulated endogenous SPRED2 in cancer, including HCC, may play a role in the regulation of cancer stem cells (CSCs).

Previous studies demonstrated the role of SPRED2 in the behaviors of cancer cells via the exogenous overexpression of this protein [23,24,25]. In the present study, we aimed to determine the biological significance of endogenous SPRED2 regarding the fate of HCC cells and demonstrate, for the first time, that the loss of endogenous SPRED2 results in increased epithelial–mesenchymal transition (EMT) and stemness, two potentially linked cellular states [26,27], in HCC cells via the upregulation of ERK1/2 and its downstream signaling pathways.

## 2. Results

### 2.1. Loss of SPRED2 Augments HCC Cell Proliferation and Invasiveness

**Figure 1:**
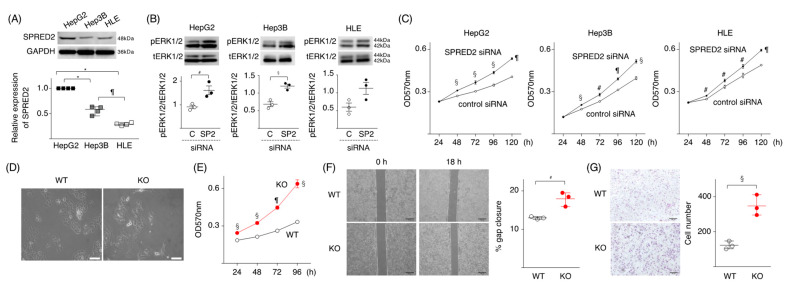
SPRED2 negatively regulates cancer cell proliferation, migration, and invasiveness. (**A**) The expression level of SPRED2 in HepG2, Hep3B and HLE cells was evaluated by western blotting. Band densities were digitized and semi-quantitated (*n* = 4, each). The level in HepG2 was regarded as 1. (**B**) ERK1/2 phosphorylation in HepG2, Hep3B and HLE, transfected with *SPRED*2-specific (SP2:●) or non-targeting control siRNA (C:○) by western blotting (*n* = 3, each). (**C**) The proliferation of each cell line was evaluated by MTT assay. (**D**) WT- and SPRED2-KO cells (2 × 10^5^/well) were incubated in 60 mm plastic dishes for 24 h and the cell morphology was examined under microscopy. Representative photos are shown. Scale bars: 50 μm. (**E**) The proliferation of WT- and SPRED2-KO cells was evaluated by MTT assay. (**F**) Migration/proliferation of WT- and SPRED2-KO cells by a scratch assay. Left panel, the images were captured with an inverted microscope. Representative photos are shown. Scale bars: 200 μm. Right panel, the gap distance at different time points was measured by Image J software (*n* = 3, each). (**G**) Matrigel invasion assay. Left panel, representative photos. Scale bars: 100 μm. Right panel, three low-power fields (magnification, 10×) were randomly selected from each chamber to count the migrated cells (*n* = 3, each). ^#^ *p* < 0.05, ^§^ *p <* 0.01, ^¶^ *p <* 0.001, * p < 0.0001, two-tailed unpaired *t* test (**A**–**C**,**E**–**G**).

We first evaluated the expression of SPRED2 in three human HCC cell lines, HepG2, Hep3B and HLE cells, with parenchymal characteristics [28]. All three cell lines expressed endogenous SPRED2, but the expression level was different among them, highest in HepG2 and lowest in HLE (Figure 1A). SPRED2-knockdown with SPRED2-specific siRNA (Figure S1) enhanced the phosphorylation of ERK1/2 (Figure 1B), and cell proliferation (Figure 1C) in all three cell lines. The effects of SPRED2-knockdown appeared highest in HepG2 cells.

To verify the results obtained by SPRED2-knockdown, we attempted to generate SPRED2-knockout (KO) cells by mutating the *SPRED2* gene in HepG2 cells by the CRISPR-Cas9 technology and obtained 7 potential clones (Figure S2). Among the 7 candidate clones, the deletion of SPRED2 protein was confirmed in two clones, B5 and E1, by western blotting. ERK1/2 activation, as evidenced by the increased phosphorylation of ERK1/2 and an increased cell proliferation, was detected in both clones (Figure S2B,C). The presence of a deletion in one allele and an insertion in the other allele in the *SPRED2* gene was detected in the genome of clone E1 by DNA sequencing (Figure S2D). The E1 clone was used in SPRED2-KO cells in subsequent experiments.

We first compared the morphological differences between wild-type (WT) and SPRED2-KO cells. WT cells showed a cobblestone epithelial morphology (Figure 1D, left), while SPRED2-KO cells displayed an elongated spindle shape with front/back polarity (Figure 1D, right), a feature of cells that undergo EMT. Functionally, the proliferation of SPRED2-KO cells was higher than that of WT cells (Figure 1E). In an in vivo transplantation model, tumors developed more frequently after the implantation of SPRED2-KO cells than of WT cells (Figure S3). In a cell scratch assay, SPRED2-KO cells showed an accelerated gap closure compared to WT cells (Figure 1F). The migration of SPRED2-KO cells was also increased in a Matrigel cell invasion assay (Figure 1G). These results strongly suggested that endogenous SPRED2 is involved in the downregulation of cell proliferation, EMT and tumorigenicity in HCC cells.

### 2.2. Loss of SPRED2 Alters the Expression of Molecules Involved in Cancer Cell Growth and Progression

**Figure 2:**
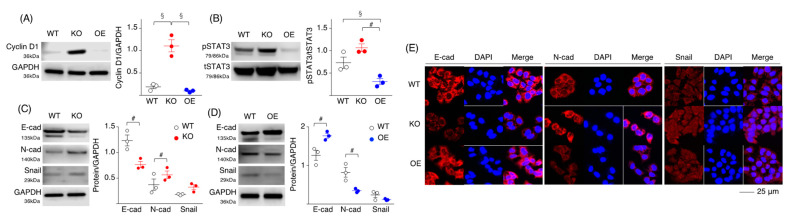
SPRED2 controls molecular expression related to cancer growth and progression. (**A**–**D**) Cell lysates were prepared from WT, SPRED2-KO and SPRED2-OE cells, and the presence of each protein was evaluated by western blotting. Band densities were digitized and semi-quantitated (*n* = 3, each). (**E**) Cells were seeded on Lab-Tek II Slide, fixed in acetone and immunostained with the indicated primary antibodies. Representative photos are shown. ^#^ *p* < 0.05, ^§^ *p <* 0.01, two-tailed unpaired *t* test.

To examine the mechanisms whereby SPRED2 regulates cell proliferation, we evaluated the effects of SPRED2-KO and SPRED2-overexpression (OE) on the activation of ERK1/2, and the expression of molecules regulating cell growth and progression. An increased SPRED2 level and a decreased ERK1/2 phosphorylation level in SPRED2-OE cells were confirmed by western blotting and immunofluorescence (Figure S4). The level of the cell cycle marker cyclin D1 was increased in SPRED2-KO cells but not in SPRED2-OE cells compared to WT cells (Figure 2A). The activation of STAT3, a transcription factor that regulates cell growth and differentiation, was also increased in SPRED2-KO cells and decreased in SPRED2-OE cells (Figure 2B).

Cadherin switching, which is defined by decreased E-cadherin and increased N-cadherin levels, is a characteristic of cells with EMT [29]. A significant decrease in the E-cadherin level and an increase in the N-cadherin level were seen in SPRED2-KO cells, whereas an increased E-cadherin level and a decreased N-cadherin level were detected in SPRED2-OE cells via western blotting (Figure 2C,D) and immunofluorescence (Figure 2E). The expression of the EMT-related transcription factor Snail was also increased in SPRED2-KO cells and decreased in SPRED2-OE cells, although the differences were not statistically significant (Figure 2C,D). Cadherin switching and augmented Snail expression were also detected in SPRED2-knockdown HepG2, Hep3B and HLE cells (Figure S5). These results further support the notion that endogenous SPRED2 plays a role in the regulation of HCC cell growth and EMT.

### 2.3. Loss of SPRED2 Increases Cancer Cell Stemness

**Figure 3:**
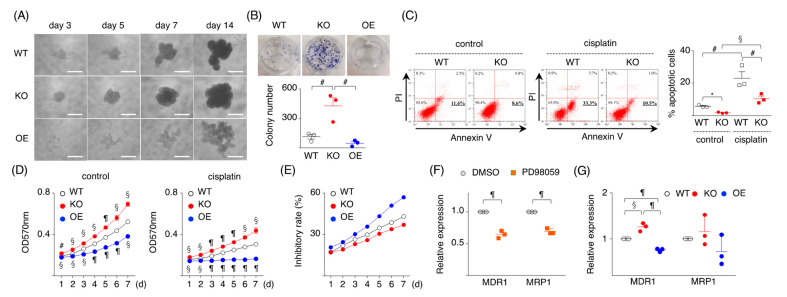
SPRED2 as a regulator of cancer cell stemness. (**A**) WT, SPRED2-KO, and SPRED2-OE cells (1000 cells) were cultured in 96-well ultra-low attachment plates for 14 days. Representative photos are shown. Scale bars: 100 μm. (**B**) Spherical colonies were dissociated, and 1 × 10^4^ cells were planted into a 6-well plate, and cultured for an additional 14 days. Upper panel, representative photos are shown. Lower panel, colony numbers in 6-well plates were counted (*n* = 3, each). (**C**) WT and SPRED2-KO cells (10^6^ cells) were cultured with or without cisplatin (10 μg/mL) for 24 h. Left panel, cells were stained with FITC-Annexin V and PI, and the percentage of apoptotic cells was analyzed by flowcytometry. Right panel, the %apoptotic cells were evaluated (*n* = 3, each). (**D**) WT, SPRED2-KO, and SPRED2-OE cells were treated with or without cisplatin (10 μg/mL) for 24 h. Cell proliferation was evaluated by MTT assay (*n* = 3, each). (**E**) Percent inhibitory rate was calculated by (1-cisplatin: A570/control: A570) ×100. (**F**) WT cells were treated with 20 μM PD98059 or vehicle (DMSO) for 24 h. *MDR1* and *MRP1* mRNA expressions in cell extracts were examined by RT-qPCR (*n* = 3, each). (**G**) The mRNA expressions of *MDR1 and MRP1* in WT, SPRED2-KO and SPRED2-OE cell lysates were evaluated by RT-qPCR (*n* = 3, each). ^#^ *p* < 0.05, ^§^ *p <* 0.01, ^¶^ *p <* 0.001, * p < 0.0001, two-tailed unpaired *t* test.

CSCs are a small subset of cancer cells that drive tumor initiation and cause relapse. Since the ERK1/2 pathway is important in the maintenance of CSCs [30] and because SPRED2-KO upregulates ERK1/2 activation, we assessed whether the loss or overexpression of SPRED2 affects the stemness of HCC cells by using a sphere formation and a spherical colony formation assay. SPRED2-KO cells formed tight stem-like spheroids faster than WT cells, while SPRED2-OE cells formed smaller spheroids with a loose structure (Figure 3A). The number of spherical colonies formed by SPRED2-KO cells was significantly higher than that by WT cells, while the number of spherical colonies formed by SPRED2-OE cells was lower relative to WT cells, although the difference was not statistically significant (Figure 3B).

CSCs are resistant to cell death and anti-cancer agents such as cisplatin [31]. We speculated that SPRED2-KO or OE may influence the sensitivity of HepG2 cells to cisplatin. The % of apoptotic cells in SPRED2-KO cells was lower than that in WT cells with or without cisplatin treatment (Figure 3C). By MTT assay, the proliferation of untreated SPRED2-KO cells was higher and conversely lower in SPRED2-OE cells compared to WT cells (Figure 3D, left). Cisplatin treatment reduced the cell proliferation of all cell types (Figure 3D, right), but chemoresistance, calculated by the mean value from each time point [(1-cisplatin:A570/control:A570) × 100], showed that the inhibitory rate was high in SPRED2-OE cells and low in SPRED2-KO cells, compared to WT cells (Figure 3E). The expression of multidrug resistance protein 1 (MDR1) and the multidrug resistance-related protein 1 (MRP1), the molecules responsible for the chemo-resistance of tumor cells [32], were next examined. The expression of both *MRP1* and *MDR1* mRNA in WT cells was significantly decreased by the MEK inhibitor PD98059 (Figure 3F), indicating that these molecules were activated through the ERK1/2 pathway. *MDR1* mRNA expression levels were increased in SPRED2-KO cells and decreased in SPRED2-OE cells, compared to WT cells (Figure 3G), although no statistical differences were found in *MRP1* expression among the cell types. These results suggest that endogenous SPRED2 may downregulate cancer cell stemness and the sensitivity to cisplatin of HCC cells by inhibiting the ERK1/2 pathway.

### 2.4. Loss of SPRED2 Upregulates the Expression of Pluripotency and Stemness Markers

**Figure 4:**
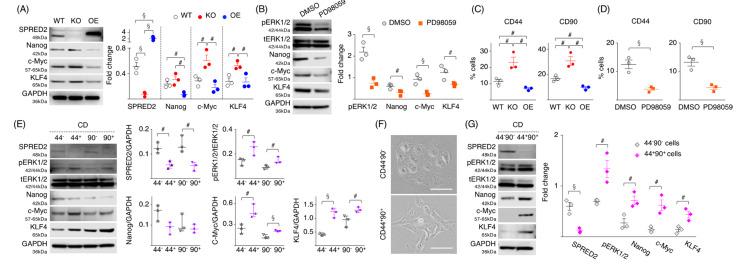
SPRED2 affects the expression of stemness markers. (**A**) Cell extracts were prepared, and the presence of each protein was evaluated by western blotting. Band densities were digitized and semi-quantitated (*n* = 3, each). (**B**) HepG2 cells were treated with 20 μM PD98059 or vehicle (DMSO) for 24 h, cell lysates were prepared, and the presence of each protein was evaluated by western blotting. Band densities were digitized and semi-quantitated (*n* = 3, each). (**C**) Cells were stained with Alexa Fluor 488-conjugated anti-human CD44 antibody or anti-human CD90 antibody, and the percentage of positive cells was analyzed by flowcytometry (*n* = 3, each). (**D**) HepG2 cells were treated with 20 μM PD98059 or vehicle (DMSO) for 24 h in standard culture conditions and stained with Alexa Fluor 488-conjugated anti-human CD44 antibody or anti-human CD90 antibody, and the percentage of CD44 or CD90 positive cells was analyzed by flowcytometry (*n* = 3, each). (**E**) CD44^+^ and CD90^+^ cells were isolated from HepG2 cells by using CD44 and CD90 microbeads. Cell lysates were prepared, and the presence of each protein was evaluated by western blotting. Band densities were digitized and semi-quantitated (*n* = 3, each). (**F**,**G**) CD44^−^CD90^−^ and CD44^+^CD90^+^ cells were isolated from HepG2 cells by using CD44 and CD90 microbeads. (**F**) Representative cell morphology is shown. Scale bars: 50 μm. (**G**) Cell lysates were prepared, and the presence of each protein was evaluated by western blotting. Band densities were digitized and semi-quantitated (*n* = 3, each). ^#^ *p* < 0.05, ^§^ *p <* 0.01, two-tailed unpaired *t* test.

To obtain further evidence supporting the suppressive role of endogenous SPRED2 in HCC cell stemness, we examined the expression of pluripotency factors that drive stemness, such as Nanog, c-Myc and KLF4 [33,34]. The loss of SPRED2 resulted in an increased expression of c-Myc and KLF4, but not of Nanog, while SPRED2 overexpression downregulated the expression of Nanog (Figure 4A). PD98059 decreased the expression of all three factors in WT cells, compared to DMSO control (Figure 4B). Although the effects by SPRED2-OE were not as potent as by PD98059, our results suggested that SPRED2 regulates the expression of pluripotency factors via inhibition of the ERK1/2 pathway.

Hepatic CSCs are reported to express several cell surface markers, including CD44 and CD90 [35,36]; therefore, we examined their expression in three cell types. The percentages of CD44^+^ or CD90^+^ cells were both higher in SPRED2-KO cells and lower in SPRED2-OE cells (Figure 4C). The percentages of CD44^+^ or CD90^+^ cells were markedly decreased by PD98059 in WT cells (Figure 4D), indicating that the expression of CD44 and CD90 in HepG2 cells was dependent on the ERK1/2 pathway. Next, we isolated CD44^−^, CD44^+^, CD90^−^ and CD90^+^ cells from WT cells and compared the expression of SPRED2 and pluripotency factors, and the phosphorylation of ERK1/2 (Figure 4E). Interestingly, the expression of SPRED2 was lower and the phosphorylation of ERK1/2 was conversely higher in CD44^+^ and CD90^+^ cells compared to CD44^−^ and CD90^−^ cells. The expression of c-Myc and KLF4 in CD44^+^ or CD90^+^ cells was high compared to CD44^−^ or CD90^−^ cells. There was no statistical difference in Nanog expression (Figure 4E).

The high expression of both CD44 and CD90 was associated with significantly reduced relapse-free survival in patients with non-small cell lung cancer [37], suggesting that CD44^+^CD90^+^ cells may have stronger CSC properties. To further evaluate the association of SPRED2 expression with CSCs, we isolated CD44^−^CD90^−^ cells (60% of total cells) or CD44^+^CD90^+^ cells (2% of total cells) from WT cells. Morphologically, CD44^−^CD90^−^ cells showed a cobblestone shape (Figure 4F, upper), whereas CD44^+^CD90^+^ cells had an elongated spindle shape (Figure 4F, lower). Interestingly, SPRED2 expression was almost undetectable in CD44^+^CD90^+^ cells, compared to CD44^−^CD90^−^ cells (Figure 4G). ERK1/2 activation and the expression of all three factors were significantly higher in CD44^+^CD90^+^ cells (Figure 4G). These results indicated a negative association between the expression of SPRED2 and stem cell markers, and suggested that endogenously expressed SPRED2 in HepG2 cells may be preventing the acquisition of stemness by inhibiting the ERK1/2 pathway.

### 2.5. SPRED2 Level Is Associated with the Stemness of HepG2 Cells

**Figure 5:**
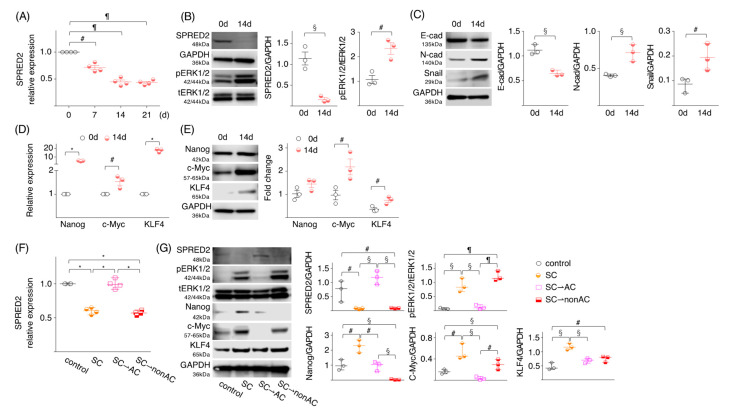
SPRED2 expression level changes under different cell culture conditions. (**A**–**C**) HepG2 cells (1000 cells) were suspended in FBS-free sphere medium, seeded in ultra-low attachment 96-well plates and incubated at 37 °C for indicated time intervals. (**A**) *SPRED2* mRNA expressions in cell extracts were examined by RT-qPCR (*n* = 3, each). (**B**,**C**) The presence of each protein in cell lysates was evaluated by western blotting. Band densities were digitized and semi-quantitated (*n* = 3, each). (**D**) mRNA expressions of stemness markers (Nanog, c-Myc and KLF4) were measured by RT-qPCR. (**E**) Protein expressions of stemness markers (Nanog, c-Myc and KLF4) were semi-quantitated by western blotting using indicated primary antibodies (*n* = 3, each). (**F**,**G**) HepG2 cells (1000 cells) were suspended in FBS (+) DMEM or FBS (−) sphere medium, and were cultured for 14 days at 37 °C in a standard 96-well plate or in an ultra-low attachment 96-well plate, respectively. A portion of cells used to seed were used as control. Cells cultured in latter conditions were divided into 3 groups. (1) Cells were continued to be cultured in a sphere-forming condition (termed, SC; sphere condition). (2) Cells were suspended in FBS (+) DMEM and cultured for an additional 3 days in a standard 6-well plate (termed, SC⇀AC; adherent condition). (3) Cells were suspended in FBS (−) sphere medium and cultured for an additional 3 days in a standard 6-well plate (termed, SC⇀nonAC). (**F**) *SPRED2* mRNA expressions in each cell extract were examined by RT-qPCR (*n* = 4, each). (**G**) Cell lysates were prepared, and the presence of each protein was evaluated by western blotting. Band densities were digitized and semi-quantitated (*n* = 3, each). ^#^ *p* < 0.05, ^§^ *p <* 0.01, ^¶^ *p <* 0.001, * p < 0.0001, two-tailed unpaired *t* test.

To further evaluate the association of SPRED2 expression and the acquisition of stemness, we cultured WT-HepG2 cells on an ultra-low attachment plate in a three-dimensional (3D) culture condition. For this, 3D tissue culture models closely resemble the natural environment of cells compared to 2D culture models, providing more physiologically useful information, which may allow for a better understanding of cancer cell biology [38]. As demonstrated above, HepG2 cells cultured in 2D expressed a significant level of SPRED2 at both the mRNA and protein level. Under a 3D culture condition, the *SPRED2* mRNA level significantly decreased with time (Figure 5A), and the level of SPRED2 protein was markedly reduced on day 14 with an increased level of ERK1/2 activation (Figure 5B). Cadherin switching and increased Snail expression were observed on day 14 (Figure 5C). The mRNA expression levels of *Nanog*, *c-Myc* and *KLF4* (Figure 5D) and the protein levels of c-Myc and KLF4 (Figure 5E) were all up-regulated on day 14, indicating that SPRED2 expression in HepG2 cells is negatively associated with the acquisition of EMT and stemness.

To examine whether 3D-cultured HepG2 cells with an increased stemness regain SPRED2 expression after incubation in 2D, spheres were washed and seeded in two standard 6-well culture plates, after which the cells were cultured in either standard medium (sphere condition: SC⇀adherent condition: AC) or serum-free sphere medium (SC⇀SC) for 3 days. The SPRED2 mRNA and protein expression levels that were reduced over 14 days in a 3D sphere culture condition (SC) returned to the original levels after 3 days of incubation in a standard adherent condition (SC⇀AC); however, they remained low in non-adherent conditions (SC⇀nonAC) (Figure 5D,E). The opposite phenomena were seen in the ERK1/2 activation, Nanog, c-Myc and KLF4 expression (Figure 5E). These results indicated that the expression of SPRED2 is negatively associated with the state of cell stemness in HepG2 cells and that this negative feedback system may no longer function in HCC cells that lose endogenous SPRED2 expression.

### 2.6. SPRED2 Expression Is Downregulated in HCC Tissues

**Figure 6:**
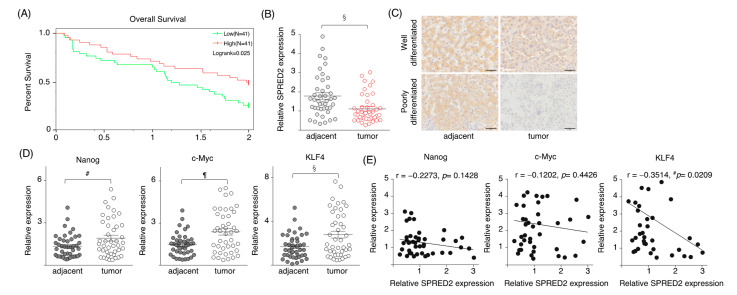
SPRED2 expression in HCC tissues. (**A**) Among 371 HCC cases from TCGA database, 82 cases that could be followed for 2 years, including those who died, were selected. The SPRED2 expression of these 82 cases was dichotomized, and a survival curve was drawn with the high 50% as SPRED2-high and the low 50% as SPRED2-low. (**B**–**E**) Data from our 40 HCC patients. (**B**) The expression levels of *SPRED2* mRNA. (**C**) Representative photos of SPRED2 immunohistochemistry from well and poorly differentiated HCC tissues. Scale bars: 50 μm. (**D**,**E**) Expressions of pluripotency factors in HCC tissues. mRNA was extracted from paraffin block. (**D**) The expression levels of Nanog, c-Myc and KLF4 were measured by RT-qPCR. (**E**) The relation between Nanog/c-Myc/Klf4 and SPRED2 was analyzed. ^#^ *p* < 0.05, ^§^ *p* < 0.01, ^¶^ *p* < 0.001, two-tailed unpaired *t* test.

Finally, we examined SPRED2 expression in the tumors of HCC patients. Among 371 HCC cases from the TCGA database, 82 cases that could be followed for 2 years, including of those who died, were selected. The overall survival of HCC patients was higher in patients with a high *SPRED2* mRNA level, compared to that of patients with a low *SPRED2* mRNA level (Figure 6A). *SPRED2* mRNA expression levels were also analyzed in 40 pairs of HCC and adjacent non-cancer tissues collected at the Okayama University Hospital (Table 1) by RT-qPCR.

The levels of *SPRED2* mRNA expression in cancer tissues were significantly lower than those in adjacent non-cancer tissues (Figure 6B). By IHC, HCC cells were stained weakly for SPRED2, whereas adjacent non-cancer hepatocytes were moderately stained. In poorly differentiated HCC tissues, the levels of SPRED2 appeared to be lower than those in well-differentiated HCC tissues, and many HCC cells were SPRED2-negative (Figure 6C). Thus, there was a negative correlation between SPRED2 levels and cancer grades. The expression levels of *Nanog*, *c-Myc* and *KLF4* in cancer tissues were significantly higher than those in adjacent non-cancer tissues in our forty pairs of HCC tissues (Figure 6D). Among these, there was a significant negative correlation between *SPRED2* and *KLF4* mRNA expression (Figure 6E), supporting a possible role of endogenous SPRED2 in the downregulation of stemness in patients with HCC. These results suggest that a loss of or a decrease in endogenous SPRED2 may contribute to the upregulation of the ERK1/2 pathway, and subsequent cancer progression in HCC.

## 3. Discussion

The expression of SPRED2 was previously shown to be downregulated in invasive carcinomas, including HCC [9,24]. The overexpression of SPRED2 inhibited SMMC-7721 HCC cell proliferation in vitro and in vivo, and induced apoptosis [24]. These results suggested that exogenously expressed SPRED2 could affect HCC cell function; however, the role of endogenously expressed SPRED2 in pathophysiology remains unknown. Here, we demonstrated that endogenous SPRED2 negatively regulated the ability of HCC cells to proliferate, migrate and invade. Endogenous SPRED2 also suppressed EMT and the acquisition of stemness, two potentially linked cellular states [26,27]. Furthermore, SPRED2 level changes in HCC cells during sphere formation in 3D culture and the SPRED2 expression levels were strongly associated with cell stemness. We previously found using a mouse bleomycin-induced lung injury model that Spred2 mRNA expression was decreased in proliferating bronchial epithelial cells (likely stem cells) after injury, but that this increased later in cells characteristic of mouse club cells, a population of stem cells that play an important role in tissue repair in the mouse airway [21]. There was a significant negative correlation between *SPRED2* and *KLF4* mRNA expression in clinical HCC tissues. Taken together, our results strongly suggested that endogenous SPRED2 may govern the biological basis of not only HCC, but also normal epithelial cells via the ERK1/2 pathway. This is the first study to suggest a functional role of endogenous SPRED2 in the regulation of cancer cell stemness.

EMT is controlled by a coordinated interplay of multiple signaling pathways [39]. There are common properties between EMT cells and CSCs, suggesting a link between EMT programs and stem cell states [26,27]. In SPRED2-KO HepG2 cells, the expression of both EMT markers and stem cell markers was upregulated. One of the pathways important for the induction of EMT is the ERK pathway [40]. STAT3 activation is another mechanism that is known to induce EMT through the induction of Snail [41]. We showed that STAT3 activation was enhanced in SPRED2-KO cells, but the direct relationship between the ERK1/2 and STAT3 signaling is unclear. Epidermal growth factor (EGF) and IL-6 activates STAT3 in different cell types, including HepG2 cells [42,43]. We found that SPRED2-KO cells, compared to WT cells, expressed a higher level of *EGF* mRNA in an unstimulated state, and showed a much higher capacity to express *EGF* and *IL-6* mRNA when stimulated (Figure S6). Thus, SPRED2 can regulate EMT directly through ERK1/2 activation and indirectly through STAT3 activation, caused by an increased cytokine production. In most types of cells, EMT leads to increased migratory and invasive properties, while reducing cell proliferation [44]. Interestingly, our results showed that the loss of SPRED2 promoted both cell proliferation and EMT, while the overexpression of SPRED2 hampered cell proliferation and EMT. We showed that STAT3 was activated by SPRED2 deficiency and was repressed by SPRED2 overexpression. This may explain our results, since STAT3 promotes cell proliferation [45], in addition to EMT induction [41]. Additional studies are required to clarify the mechanism of STAT3 activation in SPRED2-KO cells.

Uncovering the mechanism(s) that regulate cancer cell stemness continues to be a challenge. In the present study, we demonstrated evidence suggesting that endogenous SPRED2 serves as a regulator of stemness in HepG2 cells. By deleting SPRED2, HepG2 cells showed enhanced stemness phenotypes, including increases in sphere formation/spherical colony formation and the expression of stemness markers; meanwhile, the overexpression of SPRED2 demonstrated opposite effects. Interestingly, endogenous SPRED2 expression gradually decreased in HepG2 cells under a sphere culture condition without any treatment. Transforming growth factor (TGF)-β is known to induce not only EMT, but stemness characteristics [46]. We detected a decreased SPRED2 expression in HepG2 cells after treatment with TGF-β (Figure S7), suggesting that endogenous SPRED2 plays a common role in EMT and cell stemness.

Recently, it was shown that HepG2 spheroid proteome was divergent from the monolayer proteome after 14 days in culture, and that it continued to change over the successive culture time points. Not only hepatic marker proteins (e.g., albumin, α-fetoprotein), but also cell–cell interaction proteins, including cell junction, extracellular matrix, and cell adhesion proteins, were found to be continually modulated [47]. We demonstrated here a clear difference in the SPRED2 expression level and the expression of stemness markers between 2D and 3D models, suggesting the strong impact of endogenous SPRED2 on stemness regulation in HCC cells. SPRED2 is a membrane-associated substrate of receptor tyrosine kinases [48], and reacts with Raf that is localized in the raft domain of the plasma membrane [49]. The altered expression of cell–cell interaction proteins may affect the expression and function of SPRED2. It will be important to elucidate the precise mechanism that regulates the expression of SPRED2.

We have been investigating the role of SPRED2 in inflammatory responses and cancer by analyzing human data and performing experiments using human cell lines and Spred2 KO mice. However, the role of SPRED2 in the process of cell signaling is still unclear. As described above, SPRED2 inhibits the Ras-ERK1/2 signaling pathway by interacting with Raf [8]. In general, Ras is thought to be a signaling molecule that is downstream of receptor tyrosine kinases, but it also plays a role in other signaling pathways [50]. The deletion of SPRED2 likely activate many signaling pathways in which Ras plays a role. It is necessary to continue studies to better understand the role of this molecule in cancer biology.

In conclusion, we have demonstrated new evidence that strongly suggests that endogenous SPRED2 plays a critical role in the suppression of cancer cell proliferation, EMT and stemness in HCC cells. Accumulating evidence indicates that CSCs, only a rare subset of cancer cells with stem cell properties, are responsible for the early recurrence of cancer caused by tumor invasion and metastasis, as well as for the failure of chemotherapy and radiotherapy [51,52]. Therefore, targeting signaling pathways that are critical to the proliferation and survival of CSCs could present a powerful therapeutic strategy. The decreased expression of SPRED2 is associated with the grades of malignancy [9,53,54], suggesting that endogenous SPRED2 as a potential biomarker for HCC. Thus, maintaining the endogenous SPRED2 level in malignant cells could be a novel treatment strategy in order to inhibit not only cancer cell growth and progression, but also the acquisition of EMT and stemness.

## 4. Materials and Methods

### 4.1. Cell Culture

HepG2 and HLE cells (JCRB cell bank, Osaka, Japan) were cultured in Dulbecco’s modified Eagle medium (Nacalai Tesque, Kyoto, Japan), supplemented with 10% fetal bovine serum (FBS) (Gibco, Carlsbad, CA, USA), 100 U/mL penicillin and 100 μg/mL streptomycin (Sigma-Aldrich, St. Louis, MO). Hep3B cells (DS Pharma Biomedical, Osaka, Japan) were cultured in Eagle’s minimal essential medium (MEM) (Sigma-Aldrich, St. Louis, MO, USA), supplemented with MEM non-essential amino acid solution, 10% FBS, and antibiotics. In some experiments, cells were treated with the MEK/ERK1/2 inhibitor PD98059 (20 μM; Thermo Fisher Scientific, Waltham, MA, USA) or vehicle (DMSO) for 24 h. All experiments were performed with mycoplasma-free cells.

### 4.2. Transfection

Transfection was performed using Lipofectamine 3000 (Thermo Fisher) in OPTI-MEM 1X reduced serum medium (Gibco) for 48 h. For loss-of-function experiments, 2 μg of SPRED2-specific or non-targeting control siRNAs (Thermo Fisher) was introduced into HCC cells, and the cells were cultured for 48 h. For gain-of-function analysis, HCC cells were transfected with 8 μg of SPRED2 expression plasmid (Oligene, Rockville, MD, USA). The efficacy of siRNA and its overexpression were validated by real-time quantitative PCR (RT-qPCR) or western blotting.

### 4.3. Generation of SPRED2 Knockout (SPRED2-KO) Cells

HepG2 cells (2 × 10^5^ cells) were seeded into a 6-well plate. After overnight incubation, cells were transfected with SPRED2 Double Nickase Plasmid (sc-404738-NIC) or Control Double Nickase Plasmid (sc-437281) (Santa Cruz, Dallas, TX, USA) using Lipofectamine 3000 (Thermo Fisher). The sequences for sgRNAs used to disrupt the *SPRED2* gene were as follows; 5′-GCTGATGCCCGAGCCTTTGA-2′and 5′-GCAATCGAAGACCTTATAGA-3′. Then, 72 hours after transfection, the medium was changed to the same medium, which contained puromycin (2 μg/mL), and transfected cells were selected for 5 days in the presence of puromycin. Subsequently, single cell clones were selected through serial dilution.

### 4.4. Real-Time Quantitative PCR (RT-qPCR)

Total RNA was isolated from cultured cells using a High Pure RNA Isolation kit (Roche, Mannheim, Germany). First-strand cDNAs were synthesized from 2 μg of total RNA using a High-capacity cDNA reverse transcription kit (Thermo Fisher). RT- qPCR was performed using a StepOnePlus system (Thermo Fisher). The primers used in this study are listed in Table 2. The expression level of each gene was normalized against the expression level of GAPDH.

### 4.5. Western Blotting

Cells were lysed in a lysis buffer (Cell Signaling Technology). The protein concentration in the lysates was measured by BCA protein assay (TaKaRa, Kusatsu, Shiga, Japan). Equal amounts of samples (15 μg) were fractionated by sodium dodecyl sulphate–polyacrylamide gel electrophoresis (Thermo Fisher) and the proteins were transferred onto PVDF membranes. After blocking, the membranes were incubated overnight with a primary antibody, followed by a horseradish peroxidase-conjugated secondary antibody. Target proteins were visualized by ImmunoStar LD (Wako, Osaka, Japan) and the membranes were scanned using a C-DiGit Blot scanner (LI-COR Biotechnology, Lincoln, NE). The blot images were semi-quantitated with Image Studio software. The antibodies used for Western blotting are listed in Table 3.

### 4.6. Cell Proliferation and Cytotoxicity Assay

Cells were seeded in a 96-well plate at 2000 cells/well with 100 µL of cell suspension. The cell growth was determined every 24 h using the MTT assay (Roche, Mannheim, Germany). The optical density (OD) values at 570 nm were determined using a microplate reader. A higher absorbance rate indicates an increase in the cell proliferation. In some experiments, cells were exposed to a sublethal dose of cisplatin (10 µg/mL) [55]. Each assay was performed in triplicate.

### 4.7. Scratch Assay

Cells (2 × 10^5^ cells) were grown in a 6-well plate to obtain the confluence of the monolayer, scratched with a sterile 200 µL tip, and then cultured for 18 h, at a time when there was no difference in cell proliferation between SPRED2-KO and WT cells. The images were captured at different time points with an inverted microscope (Olympus CKX41; Olympus, Tokyo, Japan), and the wound distance at each time point was measured by Image J software. The percent of wound closure was assessed by (Original distance-final distance)/original distance × 100 (%). The experiments were performed in triplicate.

### 4.8. Transwell Invasion Assay

Transwell chambers (Corning, Lowell, MA, USA) were used. Cells (5 × 10^5^) were seeded on Matrigel-containing upper chamber and incubated for 18 h, at a time when there was no difference in the cell proliferation between SPRED2-KO and WT cells. Cells that invaded into the lower chamber were fixed in methanol and stained with crystal violet. Three low-power fields (magnification, ×20) were randomly selected from each chamber to count the migrated cells. The experiments were performed in triplicate.

### 4.9. Sphere Formation and Colony Formation Assay

Cells at ~80% confluence were dissociated into single-cell suspensions using 0.25% trypsin and 0.05% EDTA (Sigma-Aldrich). The cells were suspended in B-27 (Thermo Fisher); this was supplemented by DMEM/F12 medium containing 20 ng/mL of epidermal growth factor (EGF, Peprotech, Cranbury, NJ, USA) and 20 ng/mL of basic fibroblast growth factor (bFGF, Peprotech), seeded in ultra-low attachment 96-well plates (Corning) at a density of 1000 cells per well, and were incubated at 37 °C. Fresh aliquots of EGF and bFGF were added every 2 days. These were cultured for 14 days. Spheres were then dissociated, and 1 × 10^4^ cells were plated onto a 6-well plate (BioLite 6 Well Multi-dish, Thermo Fisher), cultured for an additional 14 days to investigate the self-renewal ability of the cells through secondary sphere formation. At appropriate time points, the images were captured with an inverted microscope (Olympus).

### 4.10. Fluorescence Immunostaining

Cells were seeded on a Lab-Tek II Slide (8 Chamber, Electron Microscopy Sciences, Hatfield, PA) for 1 day at 37 °C. The cells were fixed in acetone and immunostained with the indicated primary antibodies (Table 3). The slides were then incubated with Alexa Fluor 568-conjugated anti-rabbit IgG, and visualized using confocal laser scanning microscopy (LSM780, Zeiss Microscopy, Jena, Germany).

### 4.11. Flow Cytometry

For the apoptosis assay, cells were washed with PBS, resuspended in 500 µL cold PBS, and stained with fluorescein isothiocyanate (FITC)-Annexin V and Propidium Iodide (PI) using TACS Annexin V Kits (R&D Systems, Minneapolis, MN). To detect CD44^+^ and CD90^+^ cells, cells were incubated with an Alexa Fluor 488-conjugated anti-human CD44 antibody and anti-human CD90 antibody (Biolegend, San Diego, CA), respectively. Cells were analyzed using a MACSQuant Analyzer (Miltenyi Biotec, Bergisch Gladbach, Germany), and data were analyzed using MACSQuantify software (Miltenyi Biotec).

### 4.12. Cell Isolation

CD44 and CD90 Microbeads (Miltenyi Biotec) were used for the isolation of CD44^+^ and CD90^+^ cells from single-cell suspensions from WT-HepG2 cells, respectively. The purity and % of alive cells were >95%.

### 4.13. Immunohistochemistry (IHC)

Immunostaining for Spred2 was carried out using the Polink-2 plus HRP rabbit with DAB kit (GBI, Bothell, WA, USA), according to the manufacturer’s instructions. In brief, sections (4-µ-thick) were deparaffinized, rehydrated, and treated in 0.3% H_2_O_2_ in methanol for 10 min at room temperature. After antigen retrieval, using a microwave oven in 0.1 M of citric acid buffer for 25 min, sections were blocked with DAKO Protein Block Serum-Free (Dako, Carpinteria, CA, USA), and incubated with an anti-human Spred2 polyclonal antibody (Table 3) for 90 min at room temperature. After washing, sections were incubated with rabbit antibody-specific enhancer for 15 min at room temperature, followed by the incubation with polymer-HRP for rabbit IgG for 30 min at room temperature, and visualized using diaminobenzidine (Dako, Santa Clara, CA, USA). Nuclear counterstaining was performed using hematoxylin. Expression levels were determined by staining intensity.

### 4.14. Human Tissue Samples

Forty HCC surgically resected specimens were retrieved from the pathology record at the Department of Pathology, Okayama University Hospital. The patients who underwent chemotherapy or radiotherapy before the resection were not included in this study (Table 1). The protocol in this study was reviewed and approved by the Ethics Committee of Okayama University (1703-007). Although individual written consents were not obtained, we disclosed the study plan on our website, providing the patients or their families with the opportunity to opt out, and only the cases without their refusal were enrolled in the study.

### 4.15. Data Collection

A data set of 371 HCC patients was collected from The Cancer Genome Atlas (TCGA) database (https://www.cancer.gov/tcga (accessed on 6 October 2020)). Among 371 HCC cases, 82 cases that could be followed for 2 years, including those who died, were selected. The SPRED2 expression of these 82 cases was dichotomized, and a survival curve was drawn with the high 50% as SPRED2-high and the low 50% as SPRED2-low.

### 4.16. Statistics

All statistical calculations were performed using GraphPad Prism 6 (GraphPad Software, San Diego, CA, USA). Statistical significance was analyzed using a parametric two-tailed unpaired *t* test and non-parametric Mann–Whitney u test for normal distribution and non-normal distribution, respectively. Data were expressed as the mean ± SEM (normal distribution). Survival rate was compared using the log-rank test. A *p* value < 0.05 was considered statistically significant.

## Figures and Tables

**Table 1:** Cases for the enrolled HCC patients cited.

Case	Age	Sex	Stage	Growth Pattern	Tumor Grade	Cirrhosis
1	65	M	1b	mixed	moderate	−
2	71	M	1a	trabecular	well	−
3	75	F	1b	pseudoglandular	moderate	+
4	68	F	1b	mixed	poor	−
5	73	M	1b	trabecular	well	−
6	69	F	1b	trabecular	moderate	+
7	56	M	1a	trabecular	moderate	−
8	79	M	1b	mixed	moderate	+
9	61	M	2	trabecular	poor	−
10	74	M	1a	solid	moderate	+
11	79	M	2	mixed	moderate	−
12	55	M	1a	trabecular	well	+
13	82	F	2	trabecular	moderate	−
14	52	M	1a	trabecular	moderate	+
15	71	M	1b	mixed	moderate	−
16	66	M	2	mixed	well	+
17	62	M	1a	trabecular	moderate	+
18	62	F	2	trabecular	moderate	−
19	64	F	2	trabecular	well	+
20	68	M	2	mixed	moderate	−
21	59	M	1a	pseudoglandular	moderate	−
22	73	M	2	trabecular	moderate	−
23	61	F	1a	mixed	well	+
24	63	M	1a	pseudoglandular	well	−
25	73	F	1b	mixed	moderate	−
26	70	M	1b	mixed	moderate	−
27	70	M	1a	trabecular	moderate	−
28	69	M	1a	trabecular	moderate	+
29	60	F	1a	trabecular	well	+
30	62	M	1b	trabecular	moderate	−
31	57	M	1b	mixed	moderate	−
32	74	F	1a	trabecular	well	−
33	61	M	1b	mixed	moderate	−
34	64	F	1a	trabecular	well	+
35	57	F	1b	solid	poor	−
36	55	M	1b	trabecular	poor	+
37	69	M	1b	mixed	moderate	−
38	50	M	2	trabecular	moderate	+
39	77	F	2	mixed	moderate	−
40	69	F	1a	trabecular	moderate	+

Tumour stage, tumour grade and growth pattern are classified according to the WHO (2019) classification. Stage1a: Solitary tumor 2 cm or less in greatest dimension with or without vascular invasion. Stage1b: Solitary tumor more than 2 cm in greatest dimension without vascular invasion. Stage2: Solitary tumor with vascular invasion more than 2 cm dimension or multiple tumors, none more than 5 cm in greatest dimension.

**Table 2:** Taqman gene expression assays used for RT-qPCR.

Gene	Taqman Gene Expression Assay Kit
*Nanog*	Hs02387400_g1
*Myc*	Hs00153408_m1
*Klf4*	Hs00358836_m1
*MDR1*	Hs00184500_m1
*MRP1*	Hs02514106_s1
*TNF* *α*	Hs00174128_m1
*IL-6*	Hs00174131_m1
*SPRED2*	Hs00986220_m1
*GAPDH*	Hs02758991_g1

**Table 3:** Antibodies for Western blotting and immunohistochemistry.

Antigen	Company (Cat. Number)
CyclinD1	Cell Signaling Technology (92G2)
E-cadherin	Cell Signaling Technology (24E10)
N-cadherin	Cell Signaling Technology (D4R1H)
Snail	Cell Signaling Technology (C15D3)
SPRED2	Proteintech (24091-1-AP)
Nanog	Cell Signaling Technology (D73G4)
c-Myc	Cell Signaling Technology (D84C12)
KLF4	Cell Signaling Technology (4038)
GAPDH	Cell Signaling Technology (5174)
p44/42 MAPK (ERK1/2)	Cell Signaling Technology (4695)
Phospho-p44/42 MAPK (pERK1/2)(Thr 202/Tyr 204)	Cell Signaling Technology (4370)
STAT3	Cell Signaling Technology (12640)
Phospho-STAT3 (Tyr 705)	Cell Signaling Technology (9145)
HRP-goat anti-rabbit IgG	Cell Signaling Technology (7074)
HRP-anti-mouse IgG	Cell Signaling Technology (7076)

Cell signaling Technology: Denver, MA, USA. Proteintech: Rosemont, IL, USA.

## Data Availability

The data that support the findings of this study are available from the corresponding author upon reasonable request.

## Supplementary Materials

The following supporting information can be downloaded at: https://www.mdpi.com/article/10.3390/ijms24054996/s1.
