# Supplementary material for: SPRED2: A Novel Regulator of Epithelial-Mesenchymal Transition and Stemness in Hepatocellular Carcinoma Cells
Source: Int J Mol Sci. 2023 Mar 5;24(5):4996. doi: 10.3390/ijms24054996 (PMC10003366; doi:10.3390/ijms24054996)
Supplement: Supplementary file 1 [file ijms-24-04996-s001.zip › ijms-2171251-supplementary.pdf]

Supplementary data

SPRED2: A Novel Regulator of Epithelial-Mesenchymal Transition and Stemness in Hepatocellular Carcinoma Cells

Tong Gao[1], Xu Yang[1], Masayoshi Fujisawa[1], Toshiaki Ohara[1], Tianyi Wang[1], Nahoko Tomonobu[2], Masakiyo Sakaguchi[2], Teizo Yoshimura[1], and Akihiro Matsukawa[1]

[1]Department of Pathology and Experimental Medicine,

[2]Department of Cell Biology, Graduate School of Medicine, Dentistry and Pharmaceutical Sciences, Okayama University, Okayama, Japan.

Fig. S1

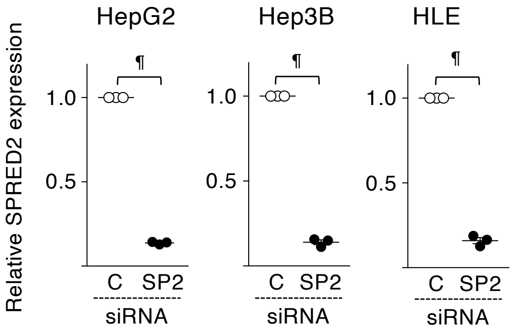

Figure S1. SPRED2 knockdown by siRNA in three different human HCC cell lines. HCC cells (HepG2, Hep3B and HLE) were transfected with 2 μg *SPRED2*-specific (SP2:●) or non-targeting control siRNA (C:○). SPRED2 mRNA expressions in HCC cells were measured by RT-qPCR. mRNA expression in each control was regarded as 1. Values are presented as the mean ± SEM. ¶p<0.001, two-tailed unpaired t test.

Fig. S2

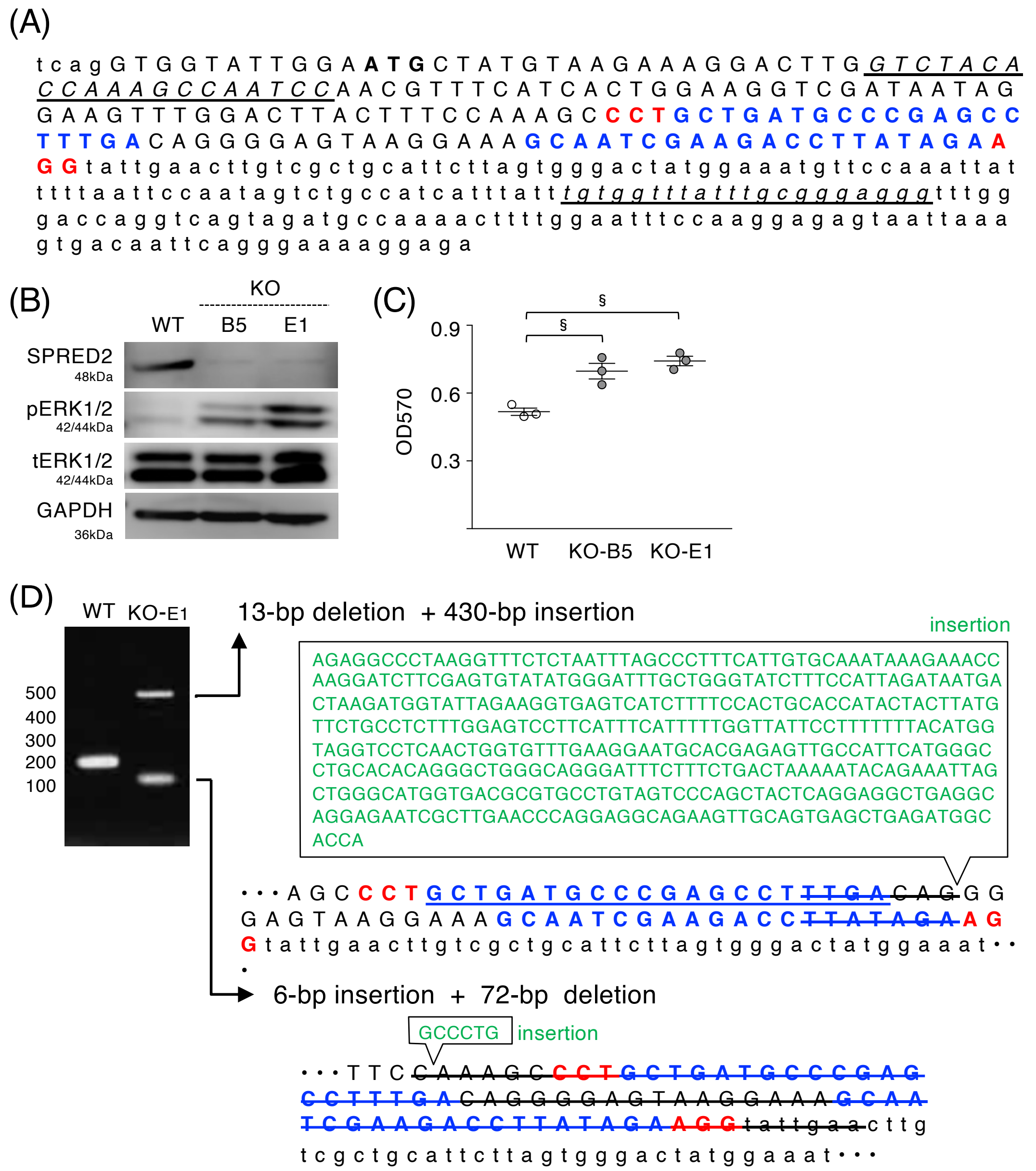

Figure S2. Generation of SPRED2 knockout (SPRED2-KO) HepG2 cells. HepG2 cells were transfected with control or SPRED2-specific Double Nickase Plasmid, and then several single cell clones were obtained. (A) The genomic sequence of the targeted region. The exon 3 sequence is in upper case and the intron 2 and 3 sequences are in lower case. The PAM sequences are indicated in red. The sequences in blue indicate the guide RNA. The sequences in <u>italic</u> indicate the primer sequences used for PCR and DNA sequencing. (B) The absence of SPRED2 protein in two SPRED2-KO clones (B5 and E1) was confirmed by western blotting. ERK1/2 activation, represented by pERK1/2 expression was enhanced in both SPRED2-KO clones as compared to WT cells. (C) Cell proliferation of SPRED2-KO clones (B5 and E1) was higher than that of WT cells. $^{§}p<0.01$, two-tailed unpaired t test. (D) Genomic DNA was isolated from WT or SPRED2-KO clone (E1) and subjected to PCR. The PCR products were analyzed by agarose gel electrophoresis and extracted from the gel and sequenced. The upper band contained a 13-bp deletion and a 430-bp insertion (in green). The lower band from SPRED2-KO clone contained a 6-bp insertion (in green) and a 72-bp deletion.

Fig. S3

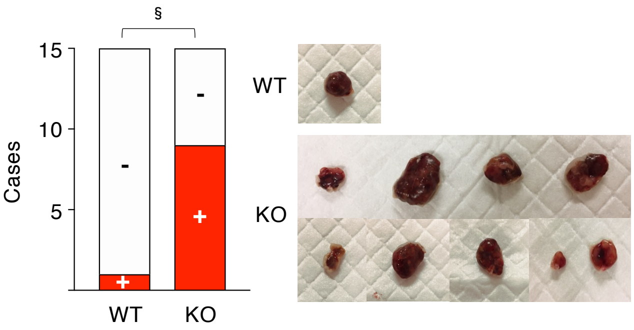

Figure S3. Tumor cell transplantation. WT and SPRED2-KO cells (5×10^6/100 μl in saline) were injected subcutaneously into the upper dorsal of 8-week-old female BALB/c nude mice (Japan SLC, Inc., Shizuoka, Japan). Eight weeks later, mice were euthanized, and the subcutaneous tumor nodules were resected when tumors were found. Tumor developed in only one out of 15 mice (6.7%) after injection of WT cells, whereas tumor developed in 9 out of 15 mice (60%) after injection of SPRED2-KO cells. $^{\S}p<0.01$, Mann Whitney u test.

Fig. S4

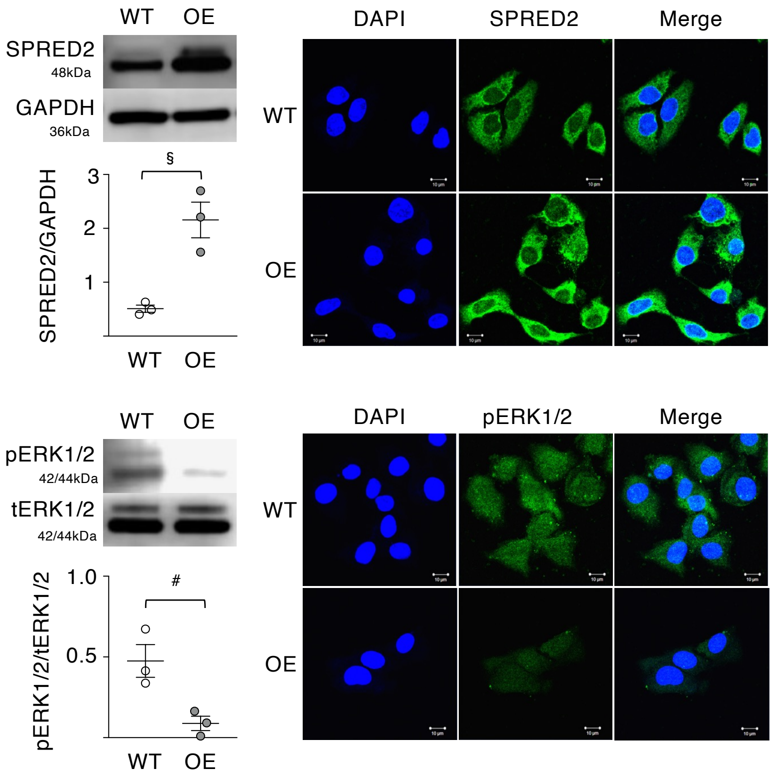

Figure S4. SPRED2 overexpression in HepG2 cells. Left, SPRED2 was transiently overexpressed in HepG2 cells (SPRED2-OE cells) by transfection of the overexpression plasmid. WT- and SPRED2-OE-HepG2 cells were extracted, immunoblotted using the indicated primary antibodies (3 each). Band densities were digitized and semi-quantitated. Values are presented as the mean ± SEM. $^{\#}$ p<0.05, $^{§}p$<0.01, two-tailed unpaired t test. Right, Cells were seeded on Lab-Tek II Slide (8 Chamber, Electron Microscopy Sciences, Hatfield, PA, USA). The cells were fixed in 95% ethanol and immune-stained with the indicated primary antibodies. DAPI was used to stain nuclear.

Fig. S5

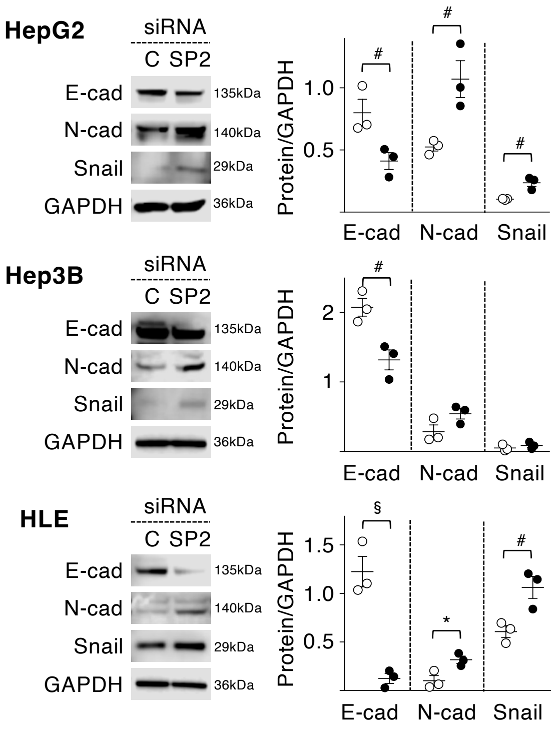

Figure S5. SPRED2 deletion in HCC cell lines. HCC cells (HepG2, Hep3B and HLE) were transfected with 2 μg *SPRED2*-specific (SP2:●) or non-targeting control siRNA (C:○). Proteins were extracted from cells and immunoblotted with the indicated primary antibodies. Band densities were digitized and semi-quantitated (n=3). $^{\#}p<0.05$, $^{§}p<0.01$, $^{*}p<0.0001$, two-tailed unpaired t test.

Fig. S6

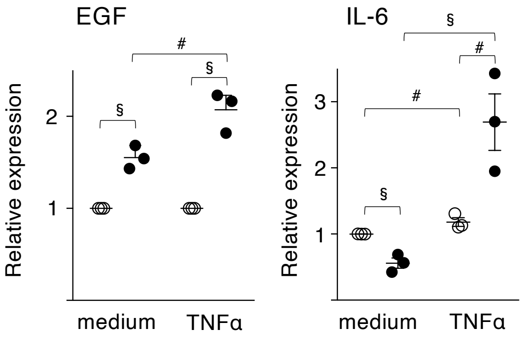

Figure S6. Expressions of EGF and IL-6 in HCC cells. WT HepG2 cells (○) and SPRED2-KO HepG2 cells (●) were stimulated with or without TNFα (100 μg/mL) for 24 hours. EGF and IL-6 mRNA expressions in cells were measured by RT-qPCR. $^{\#}p<0.05$, $^{§}p<0.01$, two-tailed unpaired t test.

Fig. S7

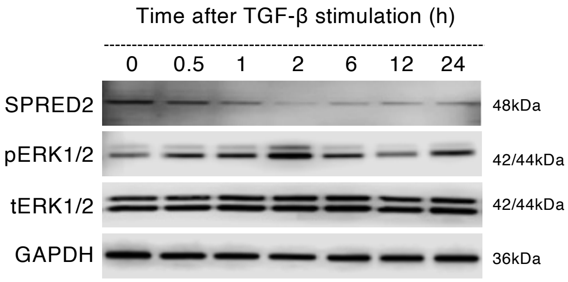

Figure S7. Expressions of SPRED2 and ERK activation after TGFβ stimulation. HepG2 cells were stimulated with TGFβ (50 μg/mL). At indicate time-point, cells were extracted, immunoblotted using the indicated primary antibodies.
